# Comparing DESI-MSI and MALDI-MSI Mediated Spatial Metabolomics and Their Applications in Cancer Studies

**DOI:** 10.3389/fonc.2022.891018

**Published:** 2022-07-18

**Authors:** Michelle Junyi He, Wenjun Pu, Xi Wang, Wei Zhang, Donge Tang, Yong Dai

**Affiliations:** ^1^ Department of Biology, Department of Brain and Cognitive Sciences, Massachusetts Institute of Technology, Cambridge, MA, United States; ^2^ Clinical Medical Research Center, Guangdong Provincial Engineering Research Center of Autoimmune Disease Precision Medicine, Shenzhen Engineering Research Center of Autoimmune Disease, The Second Clinical Medical College of Jinan University, Shenzhen People’s Hospital, Shenzhen, China; ^3^ Guangxi Key Laboratory of Metabolic Disease Research, Central Laboratory of Guilin, 924st Hospital, Guilin, China

**Keywords:** spatial metabolomics, cancer heterogeneity, breast cancer, esophageal cancer, glioblastoma, MALDI-MSI, DESI-MSI, lung cancer

## Abstract

Metabolic heterogeneity of cancer contributes significantly to its poor treatment outcomes and prognosis. As a result, studies continue to focus on identifying new biomarkers and metabolic vulnerabilities, both of which depend on the understanding of altered metabolism in cancer. In the recent decades, the rise of mass spectrometry imaging (MSI) enables the *in situ* detection of large numbers of small molecules in tissues. Therefore, researchers look to using MSI-mediated spatial metabolomics to further study the altered metabolites in cancer patients. In this review, we examined the two most commonly used spatial metabolomics techniques, MALDI-MSI and DESI-MSI, and some recent highlights of their applications in cancer studies. We also described AFADESI-MSI as a recent variation from the DESI-MSI and compare it with the two major techniques. Specifically, we discussed spatial metabolomics results in four types of heterogeneous malignancies, including breast cancer, esophageal cancer, glioblastoma and lung cancer. Multiple studies have effectively classified cancer tissue subtypes using altered metabolites information. In addition, distribution trends of key metabolites such as fatty acids, high-energy phosphate compounds, and antioxidants were identified. Therefore, while the visualization of finer distribution details requires further improvement of MSI techniques, past studies have suggested spatial metabolomics to be a promising direction to study the complexity of cancer pathophysiology.

## Introduction

Today, cancer is one of the leading causes of morbidity and mortality, resulting in around 10 million deaths worldwide each year. While new therapy and treatment methods are constantly being developed, cancer remains a challenging and deadly disease, partly due to its heterogeneous nature. For example, malignancies such as lung cancer, breast cancer, and glioma have high inter-and intra-tumoral heterogeneity and thus calls for more personalized therapy (1).

The complexity of cancer etiology and pathophysiology contributes significantly to its heterogeneity. Accumulations of genetic mutations and alterations of the epigenome are the common causes of cancer (1–3). However, environmental factors such as certain diets, obesity, and chronic inflammation also post risks to carcinogenesis (4–6). Therefore, the exact causes of the disease can be a mix of interactions between genetic and environmental factors and can vary greatly between patients. Furthermore, cancer develops an independent tumor microenvironment (TME) that consists of numerous cell types and engages in complex metabolic activities. Poor treatment outcomes are often attributed to metabolic differences between cancer cells and non-cancer cells within the TME (7). Hence, to better understand the pathophysiology of cancer and develop more effective therapies, we need to conduct studies that examine the heterogeneity of the tumor tissues.

Since one of the hallmarks of cancer is its altered metabolism, metabolomics arises as a promising direction of study. Metabolomics, or the study of small molecules, may provide a new perspective for understanding the altered interactions between the different biological pathways, enzymes, and small molecules in tumor tissues. Particularly, the emergence of spatial metabolomics offers the opportunity to detect molecules’ localizations on top of their relative abundances. This way, alterations of small molecules can be directly correlated to anatomical features. Thus it may lead to the invention of personalized medicine and faster diagnostic methods, and further understanding of heterogeneous diseases (8, 9). In this review, we summarize the recent developments of spatial metabolomics, including its common techniques (with a focus on a newer technique called AFADESI-MSI), advantages, current limitations, and some of the major findings in terms of cancer studies.

## History of Spatial Metabolomics

Despite being a relatively new field of study, metabolomics has taken several leaps forward. In 2004, Alan Saghatelian lysed cells with or without specific enzymes and examined the lysates with mass spectrometry to compare small molecule profiles (10). This approach yields metabolites that cannot be found *in vitro*. However, it was also a long and demanding process. Then, more advanced detection techniques such as liquid and gas chromatography-mass spectrometry, and nuclear magnetic resonance (NMR) enabled researchers to increase the metabolome coverage. With these techniques, studies have identified common metabolites and metabolic pathway alterations associated with various diseases such as hepatocellular carcinoma (HCC) (11), neurodegenerative disease (12), diabetes (13), *etc.* Liquid and gas chromatography, as well as NMR, offer great opportunities to discover many globally altered metabolites. However, they cannot preserve spatial information of those metabolites since they require prior extractions of the molecules from the tissues. More recently, several studies have used mass spectrometry imaging (MSI) techniques to carry out spatially resolved metabolomics, which involves examining both the chemistry and the localization of metabolites in a given tissue. Around 50 years ago, MSI was first introduced by two physicists, Benninghoven and Sichtermann, as they studied semiconductor surfaces. Then, Caprioli and co-workers combined ionization techniques with MSI and triggered a surge of growth in visualizing molecules *in situ* (14, 15). MSI techniques were first used in proteomics and lipidomic (16). However, with the rise of metabolomics, more and more studies used MSI to detect tissue distributions of key metabolites. The abnormal distributions and abundances of metabolites usually signal the alterations of biological pathways in various diseases (17). Therefore, the use of MSI in metabolomics once again increases the potentials of the field by considering the heterogeneity of the diseased tissues.

## Common Techniques for Spatial Metabolomics

Spatial metabolomics preserves metabolites’ spatial information and therefore requires simultaneous examination of large numbers of small molecules *in situ*. Most of the spatially resolved metabolomics studies coupled ionization techniques with mass spectrometry imaging (MSI) to create images of metabolites distribution. Unlike the traditional staining methods, MSI is a label-free, high-throughput technique that can detect many more molecules at once (18). As of now, most of the spatial metabolomics techniques stem from either matrix-assisted laser desorption ionization mass spectrometry imaging (MALDI-MSI) or desorption electrospray ionization mass spectrometry imaging (DESI-MSI). Both techniques are constantly being optimized. Therefore, here, we will be focusing on comparing MALDI-MSI and DESI-MSI, with a side focus on introducing one of a newer technique called air flow-assisted desorption ionization mass spectrometry imaging (AFADESI-MSI), which is based on DESI-MSI. For clearer reference, **Table 1** contrasts these three types of MSI techniques to depict their separate advantages and limitations.

**Table 1:** Contraction between MALDI-MSI and AFADESI-MSI.

MSI technique	MALDI-MSI	DESI-MSI	AFADESI-MSI
Ionization method	Matrix-assisted laser desorption ionization (MALDI)	Desorption electrospray ionization (DESI)	Air flow-assisted desorption electrospray ionization (AFADESI)
Type of MSI	Vacuum	Ambient	Ambient
Max spatial resolution	Lowest at around 1.4 µm Currently, most cancer metabolomics papers conduct experiments at around 10 µm	Lowest at 10-20 µm 50-200 µm for most of the current studies	Around 100 µm
Sample preparation	Frozen tissue or FFPE Matrix deposition	Frozen tissue or FFPE	Frozen tissue or FFPE
Key advantages	High spatial resolution and mass resolution Good for examining small samples Reliable results	High throughput Ambient operating conditions Minimum sample preparations Quick results	Ambient operating conditions Minimum sample preparations Wide field and large coverage Improve sensitivity and spatial resolution from DESI
Major limitations	Extra preparation steps Vacuum condition	Lower spatial resolution and sensitivity	Low reproducibility of results due to complex parameters

**Figure 1:**
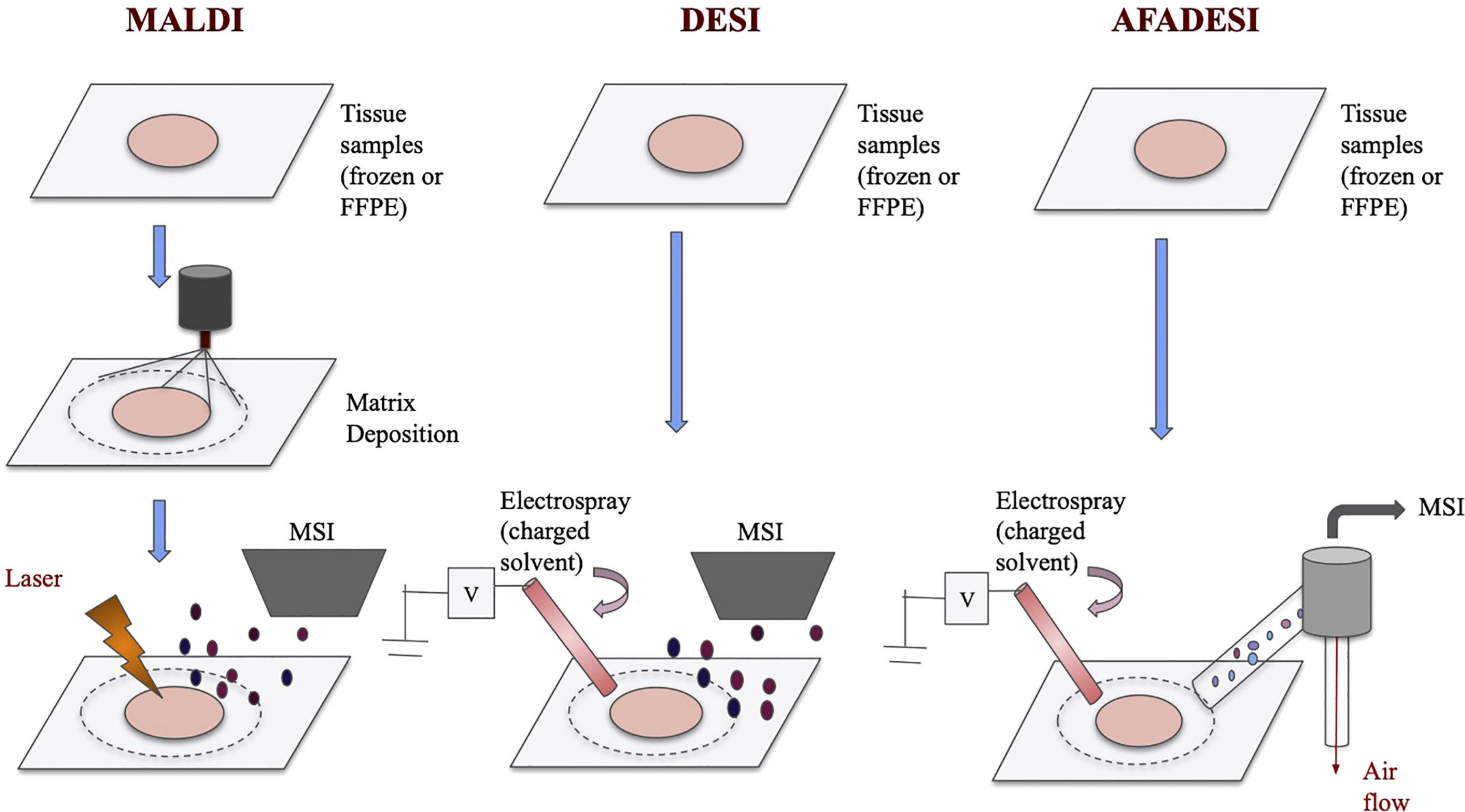
Schematics of MALDI, DESI, and AFADESI. Overview of MALDI, DESI, and AFADESI processes prior to MSI. All three techniques accept frozen or FFPE tissues, and MALDI requires an additional matrix deposition step. Subsequently, MALDI technique uses a laser to ionize the sample before MS detection whereas DESI and AFADESI use high pressure solvent to directly ionize the sample. Additionally, AFADESI depends on air flow to carry the ions over long distances to be detected.

MALDI-MSI and DESI-MSI share many similarities as they are both based on MSI to visualize metabolites information. They both have high chemical specificity and sensitivity and can detect many unlabeled analytes with relatively high spatial resolutions (19). The imaging process for both techniques, in short, involves virtually separating the sample into many “pixels,” which would each be described by a mass-to-charge (m/z) spectrum (20). Then, with the help of special analysis tools, labeled pixels that have similar metabolites signals are assigned a color and cluster together (21–23). This way, an image of the sample can be generated. Because metabolites are detected by pixels, spatial information is preserved. However, MALDI and DESI operate on different ionization principles, and therefore they each have some unique advantages. The ionization processes of these techniques are visualized in **Figure 1**.

MALDI-MSI is a matrix-dependent technique that has very high spatial resolutions. Preparation of MADI involves coating tissue samples with a low molecular weight matrix. Subsequently, a laser beam is first directed at the crystallized matrix to transform the solid matrix into a gaseous form. The energy then ionizes the sample (24). Imaging mass spectrometry is used to detect the ions and form metabolites images. MALDI is one of the most commonly used ionization methods linked to MSI as it provides a favorable balance between sample preparation, chemical sensitivity and spatial resolution (25). Now, with high-resolution MALDI-MSI, scientists can consistently detect hundreds of metabolites at a spatial resolution of <10 µm (26, 27). Spengler’s group even achieved a spatial resolution of 1.4 µm with atmospheric pressure (AP)-MALDI (28). However, as the diameter of the laser spot size decreases to achieve a finer spatial resolution, the ion yield usually decreases as well (25, 29). Therefore, currently, when conducting spatial metabolomics experiments, researchers would need to balance between having finer spatial resolutions and getting enough signal intensities. Yet, MALDI-2 has also been developed to address the sensitivity concern. With the addition of a secondary laser, MALDI-2 has improved sensitivity by 100 fold for some molecule species (25, 30). Other drawbacks of MALDI-MSI includes the decrease in resolution caused by delocation, which represents molecules diffusing across and away from the tissue. Furthermore, some papers have mentioned that MALSI-MSI can struggle in detecting low-weight molecules (<600 Da). This is because the matrix ions have similar profiles with multiple lower-weight metabolite ions and can interfere the visualizations of the metabolites (27, 31). With these limitations, there has been a growing focus on developing multimodal imaging, which means computationally imposing other imaging data with MALSI-MSI images to improve resolutions (25, 29). In the future, such approach may lead to fundamental improvement in the imaging quality of MALDI-MSI.

Another relatively newer but fast-rising technique for spatial metabolomics is DESI-MSI, which is a high-coverage technique with little pretreatment needed (32, 33). The DESI technique was introduced by Cooks’ group in 2004 and was incorporated with MSI in 2006 (34). DESI-MSI uses an ionization technique that involves directly spraying the sample with an electronically-charged solution (35). In contrast with MALDI-MSI, which is a vacuum MSI technique, DESI-MSI is the first ambient MSI technique. Ambient MSI is operationally easier and can analyze larger samples rapidly, enabling the possibility of real-time diagnosis (36–39). In addition, unlike MALDI, DESI does not require a matrix deposit. Therefore, it does not suffer from high risks of spatial assignment error caused by sample movement, which is common for the MALDI technique (40). Because of its low pretreatment requirements, DESI also allows the samples to be accessible to observation and additional processes during the analysis (35). However, improving the sensitivity of DESI-MSI has been a major challenge for the groups using the technique. Recently, a group shows that doping the solvent with silver ions may be able to improve the sensitivity and specificity of the DESI-MSI (41). Moreover, DESI-MSI also has lower spatial resolution than MALDI-MSI (30-50 µm) due to factors such as solvent composition, capillary size, and gas flow rate (42). To combat such limitations, several recent studies have used nano-DESI-MSI to achieve lower spatial resolutions (10 µm), demonstrating the potential advancements of DESI-MSI in the future (43, 44).

More recently, Abliz’s group developed a technique based on DESI called AFADESI, which significantly expands the length and area of the imaging field. AFADESI adopts the technique of DESI, ionizing the sample directly with an electrospray plume. Airflow is then used to carry ions over long distances for mass spectrometry imaging (40). In addition to inheriting the advantages of the DESI-MSI, AFADESI-MSI also can achieve a very high coverage of metabolites being examined. It can simultaneously detect thousands of molecules in an untargeted experiment (45). It also allows for the imaging of whole-body sections (46). Such coverage greatly expands the possible applications of spatial metabolomics as many more metabolites can be analyzed and compared at once. More importantly, AFADESI can achieve picomolar sensitivity when the distance between the ion transport tube and the MSI aperture is optimized (47). However, AFADESI currently has a lower spatial resolution than MALDI (around 100 µm). This would be a limitation when observing metabolites with fine localization differences. Furthermore, recent developments of AFADESI would need to focus on improving reproducibility because this technique is strongly dependent on source parameters and has relatively poor reproducibility (48). In order to improve sensitivity and reproducibility, future studies can prepare a series of uniform tissues to test out the optimal parameters and solvent compositions to be used repeatedly.

The three aforementioned techniques all have their unique strengths and weaknesses and are to be chosen based on the specific needs of the experiments. Besides these common MSI techniques, optimized ionization or data analysis methods are continually being developed to achieve higher through-put, resolution, and sensitivity. In addition, the possibility of developing multimodal imaging in the future may open up even more applications for the MSI techniques.

## Applications of Spatial Metabolomics on Cancer Studies

One of the most outstanding characteristics of tumor tissues is the alteration of the metabolic processes. Therefore, the study of spatial metabolomics has been helpful in exploring the etiology, properties, and vulnerabilities of various cancers. Previously, liquid chromatography-mass spectrometry (LC-MS) has made huge contributions in understanding key cancer metabolic pathways mediators such as the carnitine system. However, the loss of spatial information of the metabolites prevents the researchers from exploring the heterogeneity of the cancer tissues. The use of various MSI techniques in the recent studies has offered new insights into the tumor-associated metabolic reprogramming. Here, we summarized the major findings of spatial metabolomics in four types of cancer: breast cancer, esophageal cancer, glioblastoma, and lung cancer. These four types of cancers were specifically discussed because in our search for spatial metabolomics studies, most of the publications that used one of the three aforementioned techniques conducted studies on one of these types of cancer. Therefore, discussing the results for these four cancer types facilitates the comparisons between the three techniques. **Table 2** lists out the key papers we mentioned, the types of cancer and tissue they studied, the metabolites and metabolic pathways of interest, their possible clinical applications, and their methods of study.

**Table 2:** Summary of Key Papers on Spatial Metabolomics in Cancer Study.

Type of cancer	Authors	Tissue and tumor type	Key metabolites	Major ions and *m/z* values	Metabolic pathways or biological processes	Clinical relevance	Technique used
Breast Cancer:	Calligaris et al., 2014 (49)	Invasive ductal carcinoma tissues and surrounding non-neoplastic tissues	Fatty acids and lipids, especially oleic acid	Oleic acid (281.2), isobaric lipids (391.4, 655.6), PI18:0/20:4 (885.7)	G-protein coupled receptors signaling pathways; migration, proliferation, and invasion	Possible development of rapid detection of cancer residual	DESI-MSI
	Guenther et al., 2015 (50)	Invasive ductal and lobular carcinoma; tumor tissue, tumor-associated stroma, normal glandular and stromal tissue	Free fatty acids and phospholipids	Lactate 2M+Na (201.04), lactate M+Na_4_Cl_4_ (320.86), calcidiol M-2H+Na (421.32)	*De novo* lipogenesis; immune response and inflammation	Distinguish tumor grade and HR status; separate tumor-related tissues from normal tissues within samples	DESI-MSI
	Sun et al., 2020 (51)	Breast cancer tissue, normal stromal and adipose tissues	L-carnitine & acylcarnitine	L-carnitine (162.11), acylcarnitine (204.12), acylcarnitine C3:0 (218.14), C4:0 (232.15), C5:0 (246.17), C6:0 (260.19)	B-oxidation; carnitine-dependent transport system	Demonstrate carnitine reprograming in breast cancer; relate CPT 1A, CPT 2, and CRAT to altered carnitine metabolism and distribution gradient	MALDI-MSI
Esophageal Cancer:	Abbassi-Ghadi et al., 2020 (52)	esophageal adenocarcinoma and healthy esophageal epithelium tissue	glycerophospholipids	PG 36:4 (769.5025), PG 38:6 (793.5025), PG 40:8 (817.5025), PI 34:1 (835.5342),	*De novo* lipogenesis	Rapid categorization of premalignant tissues; provide possible ways for early diagnosis of the cancer and quick tumor margin detection	DESI-MSI
	Sun et al., 2019 (45)	Esophageal squamous cell carcinoma tissues (ESCC) and surrounding non-cancerous tissues	Amino acids, uridine, polyamines, fatty acids	Uracil (111.0200), histamine (112.0870), glutamate (146.0459), uridine (243.0624), FA-22:4 (331.2624), PE 36:4 (72.5146),	Amino acid metabolism (proline and glutamine), uridine metabolism, fatty acid and polyamine biosynthesis; membrane synthesis, cellular signaling, and energy consumption	Identify metabolic enzymes that are possibly involved in carcinogenesis; provide a possible way of rapidly testing large numbers of metabolites without specific targets	AFADESI-MSI
	He et al., 2018 (46)	ESCC tissue and surrounding non-cancerous tissue	polyamines, nitrogenous base, nucleoside, glutamine, carnitines, and lipids	Aspartate (132.0296), Adenine (134.0468), spermidine (146.1650), glutamate (169.0584), inosine (267.0739), adenosine (302.0669)	Polyamine catabolism, glutamine metabolism, TCA cycle	Rapidly tell apart various classes of molecules with similar masses can be helpful in specifying fine intra-regional heterogeneity	AFADESI-MSI
	Zang et al., 2021 (53)	Human esophageal cancer cell line KYSE-30 spheroid, ESCC tissue and surrounding non-cancerous tissue	Amino acids, choline, fatty acids, creatine	Creatine (132.08), malic acid (133.01), glutamine (145.06), inosine (267.07), FA 20:3 (305.25), PG 38:4 (797.53), PI 38:3 (887.56), PI 38:4 (885.55)	Fatty acid synthesis, *de novo* synthesis of choline and ethanolamine, glutamine metabolism, TCA cycle	Enable detailed study of MCTS as a cancer model; expand future usage of MCTS combined with MALDI for biomarker discovery and *in situ* drug and metabolomic study	MALDI-MSI
Glioblastoma:	Kampa et al., 2020 (54)	Glioblastoma tissue and surrounding non-cancerous tissue	Antioxidants, fatty acids, purine and pyrimidine metabolites, 2-HG, etc.	No specification of observed *m/z* Arachidonic acid (20:4), adrenic acid (22:4), oleic acid (18:1), ADP, AMPUDP, UMP, uridine, lactate, glutamine, citrate, NAA	Purine and pyrimidine metabolism, arachidonic acid synthesis, energy consumption (hydrolysis), TCA cycle	Distinguish glioblastoma subtypes; defining infiltrative tumor borders; possible use in examining therapeutic effects	MALDI-TOF-MSI
	Randall et al., 2019 (55)	Glioblastoma xenograft tissue	ATP, Heme, acylcarnitine	9-Hexadecenoylcarnitine (398.3265), palmitoylcarnitine (400.3422), myristoylcarnitine (410.2666), stearoylcarnitine (428.3734), ATP (508.0030), heme (616.1766),	Fatty acid metabolism, glycolysis; antioxidant and anti-apoptotic functions	Establish xenograft for glioblastoma therapeutic testing; understand relationship between drug efficiency and tumor metabolism	MALDI-FTICR-MSI
	Calligaris et al., 2013 (56)	Glioblastoma surgical samples that contain viable and necrotic tumor tissues	N/A	Molecules not specified. Ions with observed *m/z* of 279.0, 391.3, 544.5, 626.6, 650.6, 437.3, 491.3, 572.7	N/A	Help in real-time surgical decision-making; determine tumor border; distinguishing viable from nonviable tumor tissues	DESI-MSI
Lung Cancer:	Neumann et al., 2022 (57)	AC and SqCC tissues with tumor and stroma regions	Phospholipids, antioxidants, glutamine, 2HG	Taurine (124), [M + Cl]− ion of oxalic acid (125), 2HG (147), chloride adduct of glutamine (181), phosphatidylserine (502), phospholipid (742)	Lipogenesis, tricarboxylic acid cycle, 2HG metabolism	Distinguish tumor and stroma areas; classify ADC and SqCC subtypes for more accurate diagnosis; identify IDH mutant from wild-type cases	MALDI-MSI
	Bensussan et al., 2020 (58)	AC and SqCC tissues and FNA samples	Glycerophospholipids	FA (20:4) (303.233), PG (34:1) (747.560), PG (36:2) (773.533), PI (38:4), (788.544), PI (34:1) (835.534), PS (36:1) (885.550)	N/A	Quick discrimination of normal vs. tumor tissues for diagnosis; classification of ADC and SqCC subtypes with tissues and FNA samples	DESI-MSI

### Breast Cancer

Breast cancer is one of the most commonly diagnosed cancers in women. It is also one of the most heterogenous cancers as it consists of many different types of malignancy, originating in different cells or tissues. Hence, several groups have used MSI to visualize different aspects of metabolic alterations in various types of breast cancer. First of all, a couple of different groups used DESI-MSI to detect metabolites information and to distinguish tumor tissues from normal tissues (49, 50). In the screening, they found out that free fatty acids and phospholipids distributions played a major role in class separation. Fatty acids and phospholipids with mass greater than 760 Da have much higher levels in tumor tissues than in normal tissues. This result is consistent with our prior knowledge that cancerous cells engage in rapid lipogenesis. Using the differential fatty acids and phospholipids data in different tissue samples, Guenther and co-workers were able to correctly predict diagnosis 98.2% of the times. They found 2-10 folds increases of fatty acids and phospholipids levels from normal to diseased tissues. These results were consistent with the previous global lipidomic data conducted with LC-MS (59). Moving forward, Both Guenther and coworkers and Calligaris and co-workers exploited the unique advantage of MSI to provide spatial information on the metabolite distributions by investigating the stratification power of DESI-MSI. By examining the level of metabolites such as free fatty acid, phosphatidylcholine, lactate, and calcidiol, Guenther and co-workers classified the breast cancer samples into these six subregions: normal stroma, tumor-associated stroma, normal adipose tissue, tumor-associated adipose tissue, normal glandular tissue, and the tumor itself (neoplastic glandular cells). Each subregion can be distinguished using the metabolite information with high accuracy (the group achieved an 83.8% correct classification rate across different breast cancer types). Similarly, Calligaris and co-workers delineated the tumor margin by examining the differential intensity of representative ions such as oleic acid (m/z 281.2) and the lipid PI18:0/20:4 (*m/z* 885.7). For 12 out of 14 cases examined, the DESI-MSI data led to correct detection of the tumor margins. Hence, these studies provided a proof-of-concept experiment that confirmed the use of DESI-MSI to acquire metabolites’ spatial information in tumors. The differential metabolite distributions subsequently help the authors to classify tumor regions and types with a specificity that could not be achieved using global metabolomics data alone.

To compare the use of different ionization techniques and to investigate other key metabolites in breast cancer, we describe another group’s research below. Sun and co-workers studied the altered energy consumption of breast cancer tissues by detecting the carnitine family, which are the key regulators and transporters in fatty acid, carbohydrate, and lipid metabolisms (51). Notably, with MALDI-MSI, molecules with smaller molecular weights and lower *m/*z values were detected. Sun et al’s study examined ions over the *m/z* range of 80-1000, which is over a much larger range than that in Guenther et al.’s and Calligaris et al.’s studies (200-1000, ~700-1000, respectively). The experimental flow and clinical significance were comparable between these studies, though. First of all, as Guenther et al. have done, Sun and co-workers proved that MALDI-MSI data can help distinguish cancerous tissues from normal ones with high accuracy. They also showed that 100% ethanol wash improve the imaging of low-molecular-weight compounds such as carnitines. Using the optimized MADLI-MSI, they also found several interesting trends regarding the spatial distribution of carnitines. For example, L-carnitine and short acylcarnitine have higher levels in cancerous tissue and are strongly correlated. In addition, the abundance of L-carnitine decreases continuously as the distance from the cancer center grows. MADLI-MSI’s fine spatial resolution enabled the detection of such highly-specified, intra-regional differences in carnitine distributions. Because L-carnitine plays an important role in β-oxidation, they also examined the key enzymes of β-oxidation *in situ*. The results of enzyme distribution match the trend of L-carnitine. Such proteomics combined with metabolomics study is meaningful as it linked altered metabolites phenotypes to enzymatic changes in the biological processes. Thus, Sun and co-workers’ results not only justified the use of spatial metabolomics techniques to finely stratify cancer tissues but also reinforced our understanding of carnitines’ roles in metabolic reprogramming.

In conclusion, the studies have shown that both DESI- and MALDI-MSIs can provide valuable spatially-resolved metabolic data that help to differentiate breast cancer types and various tissue boundaries. These techniques were also able to detect a large range of metabolites and identify new biomarkers or confirm old ones based on their differential distributions. DESI-MSI demonstrated its strength in rapid, large scale detection whereas MALDI-MSI performed better in detection of smaller metabolites with finer spatial resolutions.

### Esophageal Cancer

Esophageal cancer experienced a surge in incidence during the past few decades and continues to have a poor prognosis (60). The predominant form of esophageal cancer is squamous cell carcinoma (ESCC), ranking as the top ten most common cancers (61). Another major type of esophageal cancer is adenocarcinoma (EA), which is the most common type of esophageal cancer in the US. Here, we compare spatial metabolomic studies on these two types of esophageal cancer conducted with one of the three aforementioned MSI techniques, DESI, AFADESI, and MALDI.

First, DESI-MSI has been increasingly studied as a rapid diagnostic tool for various cancers, especially in the premalignant stage. In a recent study, a group of researchers focused on spatially-resolved lipid analysis to try to identify invasive EA at an early stage from a number of premalignant tissues (52). When examined with DESI-MSI, a wide range of differentially distributed lipids were found with minimum sample preparation. With the spatial data of glycerophospholipids of mass range 600-100, the researchers could distinguish Barrett metaplasia, Barrett dysplasia, and smooth muscle within the same sample under the complex EA microenvironment. Then, comparing the EA and normal tissues, spatially-resolved lipid profiles also differ significantly that a clear distinction can be made. Particularly, the EA samples differed from the normal samples in many aspects of their glycerophospholipid profiles. First, the group found that EA samples had significantly higher levels of phosphatidylglycerol and lower levels of phosphatidylethanolamines and phosphatidic acids. Moreover, EA samples tend to have longer glycerophospholipids acyl chain length. In terms of saturation state, there are less saturated and monounsaturated acyls but more polyunsaturated ones in EA samples. Then, using principal component analysis (PCA) and recursive maximum margin criterion (RMMC) model, the group classified cancerous tissue types using phosphatidylglycerol levels, acyl chain length, and desaturation states. Upon cross-comparison with the transcriptomics data, the group also linked altered metabolites levels in EA to gene expression changes. EA had significantly higher levels of glycerophospholipid and fatty acids synthetic genes. Hence, unregulated *de novo* lipogenesis in EA is likely the cause of altered phosphatidylglycerol phenotypes. This study provided an example of using multi-omics to investigate metabolic changes in cancer tissues. Furthermore, it sheds light on a major type of metabolic reprogramming (altered lipogenesis) inside the esophageal cancer microenvironment. The successful categorization of phenotypically-similar tissue types with DESI-MSI data also demonstrated its strengths as a rapid, low-demand, wide coverage MSI technique.

However, looking to further expand DESI-MSI’s coverage and sensitivity, Abliz’s group, developed the AFADESI-MSI technique and conducted several studies on the esophageal squamous cell carcinoma (ESCC). The studies combined MSI with metabolic pathway analysis and IHC testing to investigate the reasons for regional metabolites alterations. These studies provided valuable insights onto the metabolic reprogramming of ESCC as well as the strengths and limitations of the AFADESI-MSI technique as compared to MALDI and DESI. In one study, the group used AFADESI-MSI to acquire region-specific metabolites data from 256 ESCC tissues in an untargeted experiment (45). The authors were able to prove that the AFADESI-MSI technique has high throughput and sensitivity. The detectable range of *m/z* spanned from around 100 to 1000. Moreover, multiple classes of significantly different metabolites were discovered. In terms of the specific data, fatty acid metabolism, pyrimidine metabolism, polyamine biosynthesis, and many of the amino acid metabolisms were found to be significantly dysregulated in the ESCC samples. By comparing the distributions of key synthetic enzymes, metabolic precursors, and related molecules, the group linked the upregulations of proline, glutamate, uracil, histidine, fatty acids, and polyamines to enzymatic changes and pathways dysregulations. In another study conducted by the same group, polyamines including spermine (*m/z* 203.2228) and spermidine (*m/*z 146.1650) were again found to be up-regulated in esophageal cancer tissues (46). Since spermine and spermidine regulate transcription and translation, such results may connect tumor tissues’ altered metabolism to its changes in transcriptomics and proteomics. More importantly, He and co-workers showed the sensitivity of AFADESI-MSI by finding many other classes of differentially distributed molecules with similar *m/z* ratios. For example, nucleosides including inosine (267.0739) and adenosine (302.0669) were found to be downregulated while glutamine (169.0584) was significantly upregulated. Other molecules such as L-carnitine C3:0 (218.1384) and creatine (154.0587) were also identified to be differentially distributed. Thus, AFADESI-MSI has demonstrated large coverage and high sensitivity in these two studies. These two studies also revealed the difficulties and importance of distinguishing molecules with very similar *m/z* values during spatial metabolomic studies.

However, despite all the progresses on improving the sensitivity of the DESI-based MSI, these techniques still generally have a lower sensitivity and spatial resolution. Therefore, MALDI-MSI remains the most popular method to analyze small tissue specimens. In a recent study, Abliz’s group used MALDI-MSI to examine spatial metabolomics of esophageal cancer multicellular tumor spheroids (MTCS) (53). During this study, a fine spatial resolution of 12 µm was achieved, which guaranteed visualization of spatially-resolved metabolic profiles at cellular dimensions. This way, the authors were able to examine the validity of MCTS as an esophageal cancer model and to subsequently discover metabolic trends with the established model. Interestingly, choline (104.11), glycerophosphocholine (258.11), glutamate (146.05), glutamine (145.06), xanthine (151.03), and inosine (267.07) were found to have higher concentrations in the periphery regions. Such results showed that metabolic activity is higher in the outer (proliferative) region than in the central (quiescent and necrotic) regions. Moreover, the MALDI-MSI was also able to detect trends in lipids distributions and revealed that the central regions of the tumor model engage in increased fatty acid and lipid metabolism due to hypoxia conditions. This result matched the conclusion of previous studies regarding tumor oxygen and energy usage (62–65). Further comparison between the MCTS and the human esophageal cancer tissues data revealed that MCTS is representative of the real cancer tissues. Glutamine was significantly downregulated in both the MCTS and the actual cancer tissues. It is one of the major molecular hallmarks of esophageal cancer. Creatine and malic acid were also downregulated in tumor tissues, reflecting their unchecked energy metabolism. Noticeably, the study provided the MALDI-MSI images for the cancer tissues and MCTS. Not only did the molecules show similar overall distribution trends, but the spatial distribution images of the samples under MALDI-MSI were highly comparable. Abliz’s group’s study set the example of using MTCS model combined with MALDI-MSI to investigate the detailed metabolic profiles or drug distribution patterns in tumor tissues. Lastly, it is worth mentioning that the glutamine, creatine, and malic acid distribution trends were also observed in recent studies on hepatocellular carcinoma (66, 67). Therefore, much metabolic reprogramming may be conserved across different types of cancers.

Numerous spatial metabolomics studies have been conducted on esophageal cancers, allowing us to compare the strengths and limitations of DESI-MSI, AFADES-MSI, and MALDI-MSI. DESI-MSI-derived techniques share the characteristics of having wide field and large coverage. Therefore, researchers tend to use them to develop possible rapid diagnosis and intraoperative margin detection tools. On the other hand, MALDI-MSI once again has been shown to have good spatial resolution and serves well in the examination of small biological models or samples.

### Brain Cancer (glioblastoma)

Glioblastoma, or grade 4 astrocytoma, is a common type of brain tumor and is one of the most fast-growing and aggressive cancers. It accounts for 46.1% of all primary brain malignancy incidences (68). It has some special properties as it is a central nervous system cancer and affects some unique types of cells (astrocytes and glia in the nervous system). Because glioblastoma differs significantly from most of the other types of cancer, studies have made special efforts to understand the metabolites alterations in such diseased tissues. Possibly due to the size of the common brain sample tissue and the complexity of the microenvironment, MALDI-MSI is by-far the most commonly used spatial metabolomic technique in these studies. MALDI-MSI can be coupled with different mass analyzers such as time-of-flight (ToF) and Fourier transform ion cyclotron resonance (FT ICR) to increase its mass resolution (69, 70). By doing so, these groups were able to effectively examine the highly heterogeneous glioblastoma tissues.

One study uses MALDI-Tof-MSI to compare metabolites between normal tissues and different subtypes of glioblastomas (54). The results yielded insights into the unique properties of glioblastomas. Since diffuse infiltrative growth is one of the defining characteristics of glioblastoma, histological images generated by H&E staining failed to precisely define the border of the cancerous tissues. In contrast, MSI data helped identify tumor regions by detecting differentially distributed metabolites such as antioxidants, fatty acids, and purine and pyrimidine metabolism. Interestingly, antioxidants such as taurine and ascorbic acids (vitamin C) showed decreased intensity as the distance from the tumor center grew. While the increase of taurine levels within glioblastoma tissues has been confirmed in other studies (71), the increase in ascorbic acids was detected for the first time. The high level of ascorbic acids at the tumor center was an interesting finding given that they support tumor growth and protect it from radiation. The alterations of fatty acids, lactate, purine and pyrimidine metabolites in gliomas were similar to those found in other cancers since they reflect increased energy consumption. Furthermore, the authors looked to distinguish different cancer subtypes by relying on the fine mass resolution of MALDI-Tof-MSI to detect differential metabolites. Survival rates can vary greatly between different subtypes of glioblastoma. For example, patients with isocitrate dehydrogenase (IDH) mutant gliomas have much better survival outcomes than IDH wild-type gliomas patients (72, 73). Therefore, finding ways to accurately classify the subtypes can help improve treatment and prognosis. While studying the IDH-mutant versus IDH wild-type gliomas, the group found interesting differences between the two cancer subtypes that may support previous hypotheses regarding IHC mutant pathophysiology. Specifically, besides showing higher levels of 2-hydroxyglutarate as described in many previous studies (73, 74), the IDH-mutant tumors also showed a slight decrease in antioxidants levels compared to the wild type ones. Such finding can be further explored as it matches the hypothesis that IDH mutant gliomas may be less resistant to oxidative stress, leading to better therapy outcomes (75, 76). Here, MALDI-Tof-MSI enabled clear distinction of the tumor borders and separation of the cancer subtypes as it detected metabolites differences in a highly heterogenous microenvironment. It also proved its use in distinguishing tumor subtypes using differential metabolic profiles. Therefore, the study suggested the possible usage of the same technique for analyzing therapeutics effects on glioma.

To obtain more specific spatial data for the different metabolites, a group of researchers used another improved MADLI-MSI techniques on glioblastoma models (55). Randall’s groups looked to MALDI-FTICR-MSI as an ultra-high mass resolution technique to discover finer details of metabolites distributions in tumor tissues and to test drug efficiency in relation to the tumor metabolic profiles. With the help of high-resolution imaging, Randall’s group revealed that long-chain acylcarnitine not only showed an increase in the tumor regions but were highly enriched in the tumor edge specifically. Furthermore, ATP distribution was found to have an inverse relationship with acylcarnitine distributions. Given acylcarnitine’ roles in fatty acid transportation, such finding is, again, in accordance with the previous understanding of tumor rewiring energy consumption. More importantly, the group exploited the advantages of MALDI-MSI by focusing on the spatial distribution of altered metabolites. By doing so, the group identified that there were clear metabolic changes at the edge of glioblastomas that distinguish the diseased regions from the healthy ones. Lastly, the group tested different drugs distributions and looked for their associations with the metabolites’ distributions. The drug erlotinib was found to have inverse distribution as acylcarnitine. Here, the group combine xenograft model of glioblastoma with spatial metabolomics and found new trends of acylcarnitine distributions. They were able to demonstrate the metabolic differences between the proliferative tumor edge and the tumor core. Moreover, prove MALDI-MSI to be useful in detecting spatial information of drug distribution and metabolism.

Even though MALDI-MSI has been the predominant method for studying spatially-resolved metabolic profiles of glioblastoma, a small number of studies explored DESI-MSI for glioblastoma intraoperative or xenograft model assessments. For example, Calligaris et al. did a proof-of-concept experiment to test the DESI-MSI as a real-time, invasive method to discriminate viable from nonviable tumor tissues (56). They were able to find ions that exist exclusively in viable and nonviable tissues respectively. The ions with *m/z* of 279.0 and 391.3 were discovered to be present exclusively in viable tissues whereas ions with *m/z* of 544.5, 626.6, and 650.6 were observed exclusively in necrotic tissues of the glioblastoma samples. Using the DESI-MSI data to classify regions of a tumor tissues, the authors were able to achieve very high success rate (around 98%-100%). Therefore, DESI-MSI could potentially be developed as a tool for margin delineation and tissue categorization during surgeries.

In the case of glioblastoma, MALDI-MSI serves increasingly important roles in histopathology evaluations of tissue samples and in determining biomarkers for the malignancy. DESI-MSI has more limited usage due to its lower spatial resolution. However, the high throughput imaging modality and simple preparation requirements make it useful in surgical settings.

### Lung Cancer

Lung cancer is the second-most diagnosed cancer worldwide (11.4% in 2020) and is the leading cause of cancer-related deaths (77). It also consists of numerous types of malignancies, with non-small cell lung cancer (NSCLC) being the most common one. NSCLC then contains many subtypes such as adenocarcinoma (ADC) and squamous cell carcinoma (SqCC). All of these levels of variations contribute to lung cancer’s heterogeneity. They also call for more effective diagnostic and subtyping methods for the different lung cancers. Therefore, in recent years, more and more groups started using spatial metabolomic techniques to discover new biomarkers and categorize metabolic profiles of different cancer subtypes.

One recent effort used MALDI-MSI mediated spatial metabolomics to classify the two major subtypes of NSCLC, the ADC and the SqCC, and to compare the tumor with the surrounding stroma regions (57, 78). Current IHC method fails to produce a clear histomorphological distinction between the subtypes. Therefore, Neumann and co-workers explored the distinguishing ability of MALDI-MSI. The MALDI classifier can distinguish tumor from stroma with 96% confidence and can distinguish between the two subtypes with 95% confidence. Several key ions were found to facilitate the distinction. Specifically, phospholipid (*m/z* 742) was more prominent in tumor regions compared to stroma regions. On the contrary, oxalic acid (*m/z* 125) was more prominent in stroma regions. In the subtype discriminations, m/z 124 (antioxidant taurine) and m/z 181 (chloride adduct of glutamine) showed the most different distribution patterns. In addition, m/z 502 (phosphatidylserine) had a higher intensity in ADC and thus help to distinguish the subtypes. Noticeably, during the classification, one case showed an increased intensity of 2-hydroxyglutarate (2HG) (m/z 147) in the tumor areas and was later found to be IDH mutant. Hence, the case suggested that MALDI-MSI can also possible distinguish IDH-mutated NSCLC from wild type. With the spatial metabolic profiles collected by MALDI-MSI, the researchers were able to not only classify tissues within the tumor microenvironment but also classify highly similar cancer subtypes.

Another group also attempted to classify the ADC and SqCC subtypes using DESI-MSI with fine needle aspiration samples (FNA) (58). Overall, the DESI-MSI relied on lipid and other metabolites profiles and achieved 100% accuracy on lung cancer diagnosis and 94.1% accuracy on subtyping. When using the FNA samples, the classifier achieved still an 100% accuracy on diagnosis and an 87.5% accuracy on subtyping. Interesting, the negative ion mode was examined in this study, which usually has less sensitivity and coverage. As in typical DESI-MSI experiment, a relatively high abundance of glycerophospholipids was found. PG (36:2) (773.533), PG (34:1) (747.560), and FA (20:4) (303.233) had higher intensity in normal lung tissues whereas PI (38:4) (885.550), PI (34:1) (835.534), and PS (36:1) (788.544) had higher abundance in tumor tissues. Moreover, PI (34:1) (835.534), PI (36:1) (863.565), FA (20:4) (303.233) also show significantly different distribution patterns in ADC and SqCC subtypes and therefore were used for subtype classification. For the FNA samples data, there was a decrease in the relative abundance of *m/z* 500–900 lipids comparing to that of the tissue data. Such decrease in overall detectable lipids may be the reason why FNA samples were classified with a lower accuracy. Generally, though, the two studies showed that in combination with machine learning algorithms, both the MALDI-MSI and the DESI-MSI data can be used to accurately classify NSCLC subtypes, providing a new option for more precise classification other than immunohistochemistry.

## Discussion

In recent years, spatial metabolomics has gained increasing attention as a promising way of understanding the molecular interactions and histological heterogeneity of various diseases. Previous studies have described cancer as having highly dynamic and complicated histology (79–81). Therefore, as we summarized, many cancer researchers have used MSI techniques to conduct *in situ* metabolomics on tumor tissues. MALDI and DESI remain the two most used MSI techniques for their sensitivity and reliability. The studies we examined show that under optimized conditions, MALDI and DESI produce comparable results. However, they each have their own strength and can be chosen based on the purpose of the study. Overall, the simultaneous examination of hundreds and thousands of small molecules has enabled researchers to compare metabolic reprogramming between different subtypes of tissues while discovering interesting trends regarding the localization of metabolites. However, being a relatively new field of omics research, the current spatial metabolomics study still has many limitations and unexplored aspects. For example, most papers we reviewed did not explore key metabolites’ spatial properties extensively. Characterizations of tumor subregions and identification of altered metabolites also remain limited. These results may be caused by several reasons, including limitations in MSI resolutions and sample preparations. Even though FFPE and frozen tissues are both acceptable for MSI, samples need to be prepared carefully in order to retain both the metabolites and the morphology of the tissues (8, 82). On the bright side, from MALDI-MSI to MALDI-FT ICR-MSI and from DESI-MSI to AFADESI-MSI, new techniques and research methods are continuously being developed. With the ultra-high resolution and coverage MSI, future studies can further explore spatial metabolomics of cancer tissues in several directions.

First, as the field is still relatively new, the spatial information of metabolites for many types of cancer is yet to be studied. Such metabolites data may not only help the researchers gain a comprehensive understanding of tumor pathophysiology, but also help identify new biomarkers. Many groups have been using various methods such as imaging and omics studies to discover biomarkers for personalized and targeted cancer therapies (83–85). Yet, for cancers with high inter-and intra-heterogeneity, such as lung and liver cancers, the process of examining small molecules may be challenging (86). However, lung cancer spatial metabolomics studies have mostly been subtyping studies. In the case of liver cancer, there are still a lack of metabolomics study using the three MSI techniques we mentioned. Thus, in the future, MSI-mediated spatial metabolomics can be applied to more types of cancer to compare the metabolic reprogramming between them. Researchers can benefit a lot from using MSI techniques as it offers them the opportunities to directly visualize differences in metabolites distribution and abundance within tumor tissues.

Besides expanding the use of MSI on different types of cancer, researchers can also continue exploring finer spatial differences of metabolite distributions. As of now, most papers limit their discussions on the general differences between cancerous and healthy tissues. However, with careful sample preparation and ultrahigh-resolution and sensitivity MSI techniques (87), future studies may be able to analyze new details in metabolites distribution trends. Several papers that we have reviewed explored the use of spatial metabolic data to classify different tissue subtypes and the results showed high level of accuracy. Hence, it is possible that with more detailed studies on metabolites’ localization properties, researchers can find out ways to accurately and efficiently classify tumor subregions and subtypes.

Furthermore, future studies can integrate more data and methods of study during analysis. For example, when more spatial metabolomics data on different cancer samples become available, it may be worthwhile to cross-compare metabolites distribution across multiple stages of cancer. Such comparison may reveal metabolic and physiological characteristics of cancer progression, which helps us better understand the molecular basis of cancer development and metastasis. Lastly, as the multi-omics study is now a popular topic, cross comparing transcriptomics, proteomics, and spatial metabolomics data may be a desirable direction. Spatial metabolomics data alone may have limited power in explaining the cause of metabolic alterations. However, when metabolic enzymes and their expressions are analyzed together with spatial metabolomics data, phenotypes can be linked to gene expression and protein regulation changes. This way, the metabolic pathway analysis may be more powerful and persuasive.

## Conclusion

The development of MALDI- and DESI-based MSI enables *in situ* detection of metabolites and offers great opportunities for studying disease heterogeneity and metabolic reprogramming. In cancer studies, MSI has helped identify the relative abundance and distributions of thousands of metabolites, including fatty acids, carnitines, ATP, lactate, etc. As a result, researchers attempted to use these data to classify tumor tissues and verify the current understandings of cancer metabolic interactions. Although mass and spatial resolutions remain a challenge in the development of spatial metabolomics, optimized MSI techniques are continuously being tested. The field is yet to be explored, with much potential in helping us understand cancer pathophysiology and improve diagnostic and treatment methods.

## Author Contributions

Conceptualization, MH and WP; Investigation, MH, WP, XW, and WZ; Writing-Original Draft, MH; Writing-Review and Editing, WP, DT, and YD; Supervision, DT and YD; Funding Acquisition, DT and YD. All authors contributed to the article and approved the submitted version.

## Funding

Shenzhen Fund for Guangdong Provincial High-level Clinical Key Specialties (NO.SZGSP001), Guangxi Key Laboratory of Metabolic Diseases Research (20–065–76), Shenzhen Key Medical Discipline Construction Fund (No.SZXK059), the science and technology plan of Shenzhen(NO : JCYJ20180306140810282) and the National Natural Science Foundation of China(Grant No.82003172).

## Conflict of Interest

The authors declare that the research was conducted in the absence of any commercial or financial relationships that could be construed as a potential conflict of interest.

## Publisher’s Note

All claims expressed in this article are solely those of the authors and do not necessarily represent those of their affiliated organizations, or those of the publisher, the editors and the reviewers. Any product that may be evaluated in this article, or claim that may be made by its manufacturer, is not guaranteed or endorsed by the publisher.
